# Epigenetic variance in dopamine D2 receptor: a marker of IQ malleability?

**DOI:** 10.1038/s41398-018-0222-7

**Published:** 2018-08-30

**Authors:** Jakob A. Kaminski, Florian Schlagenhauf, Michael Rapp, Swapnil Awasthi, Barbara Ruggeri, Lorenz Deserno, Tobias Banaschewski, Arun L. W. Bokde, Uli Bromberg, Christian Büchel, Erin Burke Quinlan, Sylvane Desrivières, Herta Flor, Vincent Frouin, Hugh Garavan, Penny Gowland, Bernd Ittermann, Jean-Luc Martinot, Marie-Laure Paillère Martinot, Frauke Nees, Dimitri Papadopoulos Orfanos, Tomáš Paus, Luise Poustka, Michael N. Smolka, Juliane H. Fröhner, Henrik Walter, Robert Whelan, Stephan Ripke, Gunter Schumann, Andreas Heinz, the IMAGEN consortium

**Affiliations:** 1 Department of Psychiatry and Psychotherapy, Campus Charité Mitte, Charité – Universitätsmedizin Berlin, Charitéplatz 1, 10117 Berlin, Germany; 2 Berlin Institute of Health (BIH), Kapelle Ufer 2, 10117 Berlin, Germany; 3 Max-Planck-Institute for Human Cognitive and Brainsciences, Stephanstraße 1a, 04103 Leipzig, Germany; 4 Social and Preventive Medicine, University of Potsdam, Am Neuen Palais 10, 14469 Potsdam, Germany; 5 Stanley Center for Psychiatric Research, Broad Institute of MIT and Harvard, Cambridge, MA 02142 USA; 6 Centre for Neuroimaging Sciences, Institute of Psychiatry, Psychology & Neuroscience, King’s Colleges, London, UK; 7 Department of Child and Adolescent Psychiatry, Psychotherapy and Psychosomatics, University of Leipzig, Liebigstrße 20a, 04103 Leipzig, Germany; 8 Department of Child and Adolescent Psychiatry and Psychotherapy, Central Institute of Mental Health, Medical Faculty Mannheim, Heidelberg University, Square J5, 68159 Mannheim, Germany; 9 Discipline of Psychiatry, School of Medicine and Trinity College Institute of Neuroscience, Trinity College Dublin, Dublin, Ireland; 10 University Medical Centre Hamburg-Eppendorf, House W34, 3.OG, Martinistr. 52, 20246 Hamburg, Germany; 11 Medical Research Council—Social, Genetic and Developmental Psychiatry Centre, Institute of Psychiatry, Psychology & Neuroscience, King’s College, London, UK; 12 Department of Cognitive and Clinical Neuroscience, Central Institute of Mental Health, Medical Faculty Mannheim, Heidelberg University, Square J5, Mannheim, Germany; 13 Department of Psychology, School of Social Sciences, University of Mannheim, 68131 Mannheim, Germany; 14 Neurospin, Commissariat à l’Energie Atomique, CEA-Saclay Center, Paris, France; 15 Departments of Psychiatry and Psychology, University of Vermont, Burlington, VT 05405 USA; 16 Sir Peter Mansfield Imaging Centre School of Physics and Astronomy, University of Nottingham, University Park, Nottingham, UK; 17 Physikalisch-Technische Bundesanstalt (PTB), Abbestr. 2-12, Berlin, Germany; 18 Institut National de la Santé et de la Recherche Médicale, INSERM Unit 1000 “Neuroimaging & Psychiatry”, University Paris Sud, University Paris Descartes—Sorbonne Paris Cité, Maison de Solenn, Paris, France; 19 Institut National de la Santé et de la Recherche Médicale, INSERM Unit 1000 “Neuroimaging & Psychiatry”, University Paris Sud, University Paris Descartes—Sorbonne Paris Cité; and AP-HP, Department of Adolescent Psychopathology and Medicine, Maison de Solenn, Cochin Hospital, Paris, France; 20 Rotman Research Institute, Baycrest and Departments of Psychology and Psychiatry, University of Toronto, Toronto, ON M6A 2E1 Canada; 21 Department of Child and Adolescent Psychiatry and Psychotherapy, Medical University of Vienna, Vienna, Austria; 22 Department of Psychiatry and Neuroimaging Center, Technische Universität Dresden, Dresden, Germany; 23 Department of Psychology, University College Dublin, Dublin, Ireland

## Abstract

Genetic and environmental factors both contribute to cognitive test performance. A substantial increase in average intelligence test results in the second half of the previous century within one generation is unlikely to be explained by genetic changes. One possible explanation for the strong malleability of cognitive performance measure is that environmental factors modify gene expression via epigenetic mechanisms. Epigenetic factors may help to understand the recent observations of an association between dopamine-dependent encoding of reward prediction errors and cognitive capacity, which was modulated by adverse life events. The possible manifestation of malleable biomarkers contributing to variance in cognitive test performance, and thus possibly contributing to the “missing heritability” between estimates from twin studies and variance explained by genetic markers, is still unclear. Here we show in 1475 healthy adolescents from the IMaging and GENetics (IMAGEN) sample that general IQ (gIQ) is associated with (1) polygenic scores for intelligence, (2) epigenetic modification of *DRD2* gene, (3) gray matter density in striatum, and (4) functional striatal activation elicited by temporarily surprising reward-predicting cues. Comparing the relative importance for the prediction of gIQ in an overlapping subsample, our results demonstrate neurobiological correlates of the malleability of gIQ and point to equal importance of genetic variance, epigenetic modification of DRD2 receptor gene, as well as functional striatal activation, known to influence dopamine neurotransmission. Peripheral epigenetic markers are in need of confirmation in the central nervous system and should be tested in longitudinal settings specifically assessing individual and environmental factors that modify epigenetic structure.

## Introduction

Genetic variance is known to explain a substantial part of variability in cognitive capacity^1–5^. The largest available study describes that polygenic scores (i.e., those common genetic variants that are most strongly associated with test performance in previous studies) explain up to 4.8%^4^ of the variance of general intelligence quotient IQ (gIQ). A more recent larger but not yet peer reviewed study, shows up to 5.4% of variance explained^5^. On the other hand, environmental factors have a significant impact on general cognitive capacity, as indicated by the strong rise in average IQ performance following the decades after World War II^6,7^. According to Flynn et al.^7^ the change ranged from 5 to 25 IQ points (eg. 0.3 to 1.7 standard deviation (SD)) within one generation. This change appears to be too strong to be explained by genetic changes. While various environmental factors (e.g. changes in the educational system, overall stress experience, nutrition, etc.) might contribute to this so-called Flynn effect, those factors should act via changes in neurobiological systems relevant for cognition. Possible neurobiological factors that mediate this effect and link genotype with complex traits like cognition are (1) epigenetic markers including methylation count, (2) cortical architecture of the brain evaluated using magnetic resonance imaging (MRI), and (3) the functioning of the brain explored in vivo with functional MRI (fMRI). Those malleable markers might as well contribute to the “missing heritability” that is present between variance explained by accumulating single-nucleotide polymorphisms (SNPs; 4.8% based on polygenic scoring^5^), estimates of genomic similarities between individuals (~20% SNP heritability^5^), and based on heritability estimates from twin studies (50–70%^2,3^). Here we aim to explore individual variance in gIQ that can be accounted for by neurobiological markers of cognitive performance and describe the interplay of mechanisms, including epigenetic variance that may contribute to individual malleability in cognitive capacity.

Several lines of evidence suggest that gIQ is associated with the architecture of the brain measured as cortical volume and thickness^8^ explaining up to 16% of the variance in right insula. Beyond cortical findings, the architecture^9^ and volume of subcortical structures have been associated with cognitive capacity explaining between 2.4% in striatum^10^ and up to 4.2% in caudate volume^11^. The importance of subcortical structures is further underpinned by the finding that training in reasoning alters resting state connectivity between subcortical and cortical brain areas, including striatum, parietal, and prefrontal areas^12^. This is highly plausible given the relevance of cortico-striatal networks implicated in executive function and goal-directed behavior^13–15^. In line with this, dopamine synthesis capacity in the ventral striatum has been associated with frontal cortical and striatal functional activation during goal-directed vs. habitual decision-making as well as IQ^16,17^, in accordance with the well-known role of dopamine in cognition and decision-making^18–20^. A readily available proxy for dopaminergic neurotransmission is the well-known reward anticipation signal that can be measured with fMRI^21,22^. Dopaminergic neurotransmission is partly heritable, but also substantially modulated by environmental factors^23–27^. An emerging field that could potentially link the abovementioned environmental factors and dopaminergic neurotransmission is epigenetic modulation, which can help to explain individual malleability. Finding possible links between epigenetic changes, reward signaling, and cognitive capacity in adolescents might contribute further evidence for long-lasting neurobiological correlates of environmental effects, including stress exposure, as already observed in rodents (for a review see Meaney et al.^28^).

The aforementioned candidate markers for neurobiological underpinnings of cognitive capacity have been assessed before^29^, however, their relative importance has not been tested in a cumulative fashion. Moreover, the interplay between genetic variance and possible neurobiological underpinnings of individual difference in cognitive capacity, including epigenetic markers is not known in detail. Data from the IMaging and GENetics (IMAGEN) consortium, offer a well-characterized sample to study these topics. With experts from a variety of fields, we aimed at contributing a broader insight into that research question.

Therefore, we measured associations between cognitive capacity (gIQ) and polygenic scores, epigenetic markers of the dopaminergic system, gray matter density in striatum, and striatal activation during reward processing, in a large sample of healthy adolescents, and we quantified their relative contribution to interindividual differences in IQ. We addressed the following research questions:

1. Do two different polygenic scores, which have previously been associated with cognitive capacity^4,30^, replicate in our sample?
2. Are there epigenetic markers (i.e., methylation count) of the dopaminergic system that show associations with gIQ?
3. Can we replicate previous findings^9–11^ of a correlation between gray matter density in bilateral striatum and gIQ?
4. Can the previously observed association between functional activation of the ventral striatum (BOLD-signal) and IQ^17,31^ be replicated in a large sample of adolescents?

In a subset of individuals for whom we have complete data, we evaluated the relative contribution of each of the aforementioned predictors for gIQ, assessed possible interactions of genetic variance with our other predictors, and performed model comparison for combinations of predictors.

## Materials and methods

### Participants

We used a sample of 1475 adolescents (mean age = 14.43 years; SD = 0.45, 765 female participants) from the large multicenter imaging and genetics study (IMAGEN^32^) with available data from neuropsychological assessment, functional imaging, and genetic data. The study is intended to investigate the genetic and neurobiological basis of individual variability in psychological traits, and their relation to the development of frequent neuropsychiatric disorders. Recruiting took place at eight different sites (Germany, United Kingdom, France, and Ireland). We therefore included site as a covariate in all analysis in order to account for variance introduced by center-specific variations. We excluded subjects with contraindication for MRI scans as well as serious medical conditions. Each local ethics committee approved the study. Subjects and their parents provided informed assent and consent, respectively.

### Intelligence measure

In previous work we started out with a focus on the fluid and crystallized IQ and stress exposure^17,31^. An abundant body of work on cognitive capacity and neurobiological correlates is based on a general factor derived from principal component analysis (PCA)^29^. On the other hand there is considerable criticism of constructing general factors^33^ regarding cognitive test performance and the authors have voiced similar concerns elsewhere^34^. PCA gains general information at the expense of specific information associated with the Wechsler’s intelligence scale (WISC) IV subscales. Calculating a general factor based on PCA (for a review see Deary et al.^29^) does not necessarily invalidate the nature of the original scales; instead, dimensionality reduction allows for capturing variance that is common to a variety of subscales. In this study, we therefore performed PCA in order to derive a measure of general cognitive ability from WISC IV subtests, comprising matrix reasoning, block design, digitspan backward and forward, similarities, and vocabulary^35^. The first principal component (gIQ) explained a large proportion of the variance (variance explained = 0.49) and was used for further analyses as a marker for gIQ (see Table 1 in the supplement). For a more fine-grained view we explored WISC IV subscales associations with biological markers calculating a correlation matrix in an overlapping subsample (see supplementary Table 7).

**Table 1:** Correlation matrix for predictors of overlapping sample of *n* = 755

	gIQ	BOLD	Epigenetic	Polygenic score
BOLD	0.14****			
Epigenetic	−0.10**	0		
Polygenic score	0.13****	−0.03	−0.03	
Gray matter	0.03	0	−0.03	0.01

The correlation coefficients are based on linear regressions on residuals partialling out variance from variables of no interest

*gIQ* general IQ

BOLD, functional activation during reward anticipation; epigenetic, methylation in CG site *DRD2* cg26132809; polygenic score, polygenic score including 5636 SNPs significant at a *p*-threshold of 0.01 from Sniekers et al.; gray matter, gray matter density in striatum

Significant levels (two-tailed) ****p < 0.0001; ***p < 0.001; **p < 0.01, *p < 0.05

### Genetics

For building a polygenic score, we obtained summary statistics from two large genome-wide association studies: Benyamin et al.^30^ report associations with childhood intelligence^30^ on 17 989 individuals and 1 380 159 SNPs; Sniekers et al.^4^ provide a meta-analysis and report associations of common variants with intelligence in a maximum of 78 308 adults and children and included 10 499 625 SNPs.

We performed linkage disequilibrium (LD) pruning and “clumped” the summary statistics, discarding variants within 500 kb of, and in *r*^2^ ≥ 0.1 with, another (more significant) marker. After pruning we had 70 568 LD-independent SNPs for the score by Benyamin et al.^30^ and 86 330 for the score according to Sniekers et al.^4^. For both scores we performed risk profile scores (RPS) of our sample described for a range of *p*-value thresholds (5 × 10^−8^, 1 × 10^−6^, 1 × 10^−4^, 0.001, 0.01, 0.05, 0.1, 0.2, 0.5, and 1.0), multiplying the logistic regression (i.e., the natural log of the odds ratio) of each variant by the imputation probability for the “risk” allele in each individual. The resulting values were summed over each individual, so that each individual had a whole-genome RPS for further analysis. We aimed at replicating the association of the polygenic score with gIQ in a sample of 1388 subjects with sufficient data quality.

### Epigenetics

Global blood DNA methylation levels were assessed by hybridizing DNA samples to the Infinium Human Methylation 450 Bead Chip (Illumina: http://www.illumina.com/products/methylation_450_beadchip_kits.html), following the manufacturer’s protocol. Unlike polygenic scores based on multiple SNPs, no epigenetic score exists for intelligence. With respect to epigenome-wide data, our sample size is far too small to find effects on an epigenome-wide association study (EWAS) level. Therefore, we focused on epigenetic markers potentially affecting dopamine-dependent neural encoding of reward anticipation in the striatum and planned Bonferroni correction for multiple testing.

The FDb.InfiniumMethylation.hg19 package for R (https://bioconductor.org/packages/release/data/annotation/manuals/FDb.InfiniumMethylation.hg19/man/FDb.InfiniumMethylation.hg19.pdf) was used to tag candidate gene to its nearest CG site. We extracted the start coordinate for our candidate probes from this package. This start coordinate was then used to go up and down 50 kb to create a region file. We assessed methylation count in CG site from the following genes involved in dopamine metabolism and neurotransmission: tyrosine hydroxylase (*TH*); DOPA decarboxylase (*DDC*); catechol-*O*-methyl transferase (*COMT*); dopamine transporter 1 (*SLC6A3*); dopamine receptor D1 (*DRD1*); and dopamine receptor D2 (DRD2) resulting in 24 CG sites. We focused on D1 and D2 receptors because they are the most abundant dopamine receptors in the brain with expression in regions relevant for motor, limbic, and neuroendocrine functioning^36^. D3, D4, and D5 mRNAs are one to two orders of magnitude lower than that of the D1 or D2^37^. We think that in addition to D1 and D2, D3, 4, or 5 only provide limited further insight for possible markers of gIQ. Nonetheless, for a more comprehensive view, we include an exploratory search for D3, D4, and D5 receptor gene and tested for association with gIQ in the Supplement. Epigenetic data with sufficient quality and corresponding data on gIQ was available for 817 subjects.

### Magnetic resonance imaging

#### Structural MRI

Subjects were scanned in 3T-MRI-Scanners from different manufacturers (Bruker, General Electric, Philips and Siemens^32^). We controlled for variance accounted for by scanning site using dummy coded variables^38^. We used high resolution T1-weighted three-dimensional magnetization prepared rapid gradient echo sequence based on the ADNI protocol (http://www.loni.ucla.edu/ADNI/Cores/index.shtml). Gray matter density was estimated, including age, gender, and total intracranial volume as covariates of no interest. Mean striatal gray matter density was extracted from anatomical masks using the WFU-Pick atlas^39^ comprising bilateral striatum (http://fmri.wfubmc.edu/software/pickatlas) as an anatomic voxel mask with the individual Brain Atlases tool in SPM (IBASPM 71). Structural imaging data with sufficient quality and corresponding data on gIQ were available for 1401 subjects.

#### Task details

During fMRI subjects performed a modified version of the well-known monetary incentive delay (MID) task^22,40^. The MID assesses how quickly the subject can react to a reward-indicating vs. neutral cues and pull a trigger to hit a target (with the left or right index finger). The cue was followed by a variable anticipation interval. Then the subjects were asked to push a button with their left or right index finger in order to hit an appearing target. If the subject is able to hit the target, following a reward-indicating (but not a neutral) cue, he or she scores points (Fig. 1 in SI).

#### Functional MRI

Due to our a priori hypotheses of an association between intelligence measures with activation of the ventral striatum during reward anticipation in the MID task^22^, we tested our research question in a region of interest (ROI). For this a literature-based mask^41^ of the bilateral ventral striatum was used in order to test for effects of individual signal change. We extracted the mean beta-values from the main effect in the abovementioned volume of interest of the ventral striatum from contrast images estimating the BOLD-signal change during anticipation of big and small vs. no reward (Table 5 in SI). This signal is considered as an estimate of temporal difference errors elicited by temporarily surprising reward-predicting cues, which are related to phasic dopaminergic neurotransmission (Fig. 1 in SI)^22,42^. For further details concerning scanning parameters, preprocessing, and single subject statistics please refer to the Supplement.

#### Statistical analysis

We assumed that for a sufficient power of 1 − *β* = 80% (*α* = 5%) and a small effect sizes ranging from 2.4%^10^ in previous structural imaging studies and 4.8%^4^ for previous polygenic scores, according to Hulley et al.^43^ we would need a total sample size of 161–324 subjects. To estimate the variance explained by two different polygenic scores in our sample, we were able to calculate linear regression models with gIQ as dependent variable and polygenic scores as predictors in *n* = 1388 subjects. Additional covariates included age, gender, and principal components from our prior PCA, which account for population stratification and tested two different polygenic scores, therefore we chose a significance level of *p* = 0.025.

**Fig. 1:**
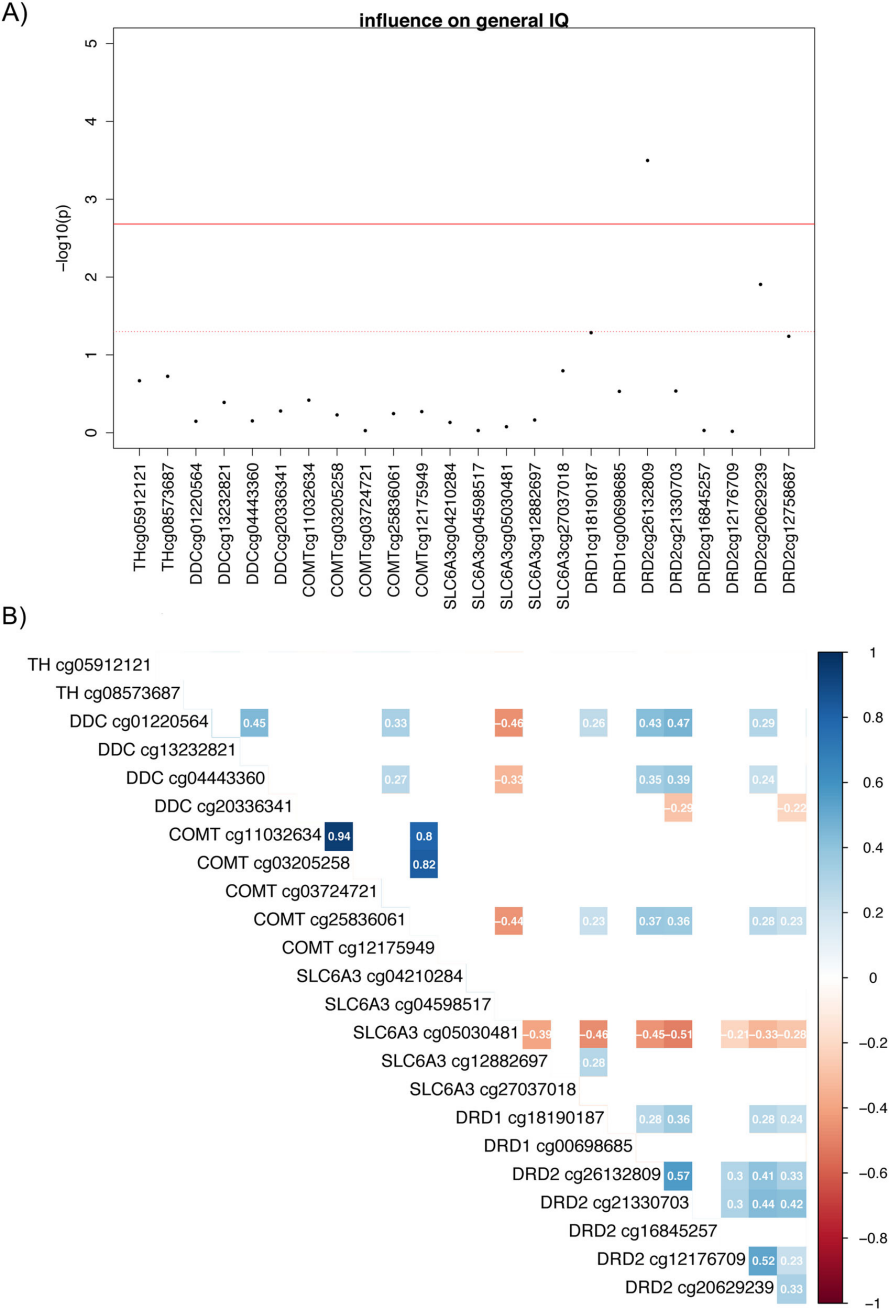
Association between methylation count in dopaminergic candidate markers and general IQ in *n* = 817 subjects. **a** Plot of negative decadic logarithm of *p*-values for association of methylation count in CG site 50 kb pairs up- and downstream from dopaminergic candidate markers. Candidate markers were tyrosine hydroxylase (*TH*), DOPA decarboxylase (*DDC*), catechol-*O*-methyl transferase (*COMT*), dopamine transporter 1 (*SLC6A3*), dopamine receptor 1 (*DRD1*), and dopamine receptor 2 (*DRD2*). Among 24 identified CG sites we found significant associations of epigenetic candidate markers for dopamine D2 receptor (cg26132809) involved in dopamine neurotransmission with general IQ correcting for age, gender, study site, wave information, and variability in cell type. The red line marks *p*-value threshold for multiple comparison correction for each CG site (*p* < 2 × 10^−3^) and the dashed line for *p* < 0.05. **b** Correlation matrix of epigenetic candidate markers involved in dopaminergic neurotransmission. Only correlation indices are displayed at a significance level of *p* < 0.01 (i.e., *R* > 0.2). Correlation coefficients are color-coded (positive correlation blue, negative correlation red)

For epigenetic markers, linear regression models were fitted for each marker in a combined sample of 817 subjects. Age, gender, and site as well as first two principle components of estimated differential cell counts and wave information were included into linear regression models as variables of no interest. We plotted a correlation matrix for all candidate markers in order to explore associations between methylation count in each CG site. Correlations between candidate CG sites revealed that most regions were independent markers (Fig. 1b). As candidate markers appeared to be rather independent, we decided to apply Bonferroni correction to rigorously correct our results for multiple comparisons resulting in a significance level of *p* = 2 × 10^−3^.

We applied linear regression to estimate the correlation between gIQ and bilateral gray matter density in striatum of 1401 subjects with sufficient imaging quality. We accounted for variance from the following variables of no interest: age, gender, site, and total brain volume.

To statistically evaluate associations between bilateral ventral striatal reward anticipation signal (BOLD-signal) and gIQ, we used multiple linear regression controlling for age, gender, and site in a sample of 1475 subjects. For imaging parameters, we used split-half cross-validation on two subsets. A significance level of *p* = 0.05 was chosen.

For explorative analysis of gIQ and whole-brain associations with BOLD-signal during reward anticipation, we computed linear regression models at each voxel, using ordinary least squares. Due to spatial auto-correlation we used whole-brain family-wise error correction (*p* = 0.05) applying random field theory as implemented in statistical parametric mapping software (SPM 8) in *n* = 1475 subjects.

For our best predictors, we estimated variance explained and obtained 95% bootstrapped confidence intervals from 1000 randomly drawn samples to evaluate reliability of our results.

For further analysis, we choose to partial out variance from variables of no interest by calculating separate regression models of our nuisance variables on our predictors. For the polygenic scores we regressed out variance accounted for by age, gender, and principal component from our prior PCA. For epigenetic markers, we accounted for age, gender, site, first two principle components of estimated cell count, and wave information. For structural MRI, we regressed out variance from age, gender, site, and total brain volume. For fMRI, we accounted for age, gender, and site. To explore possible interrelatedness between residuals of our variables, we calculated a correlation matrix.

We calculated one multiple linear regression model on residuals of our predictors in an overlapping subsample of 755 subjects with gIQ as independent variable and BOLD-signal, gray matter density, polygenic score, and epigenetic candidate marker as predictors. In order to estimate the effect size, we calculated standardized parameter estimates (beta) in a multiple regression model, which assumes standardized predictors and dependent variables (variance equals one). The standardized parameter estimate (beta) indicates how many SDs gIQ will change, per SD change in the predictor variable. We used the lavaan package^44^ in combination with the SemPlot package^45^ in R 3.2.4 for illustration purposes.

Although we did not primarily hypothesize interaction effects, we calculated interaction terms in order to explore the interplay between our variables. We focused on possible interactions of genetic effects on epigenetic, structural MRI and BOLD-signal resulting in three interaction terms (gene × epigenetic marker, gene × structural MRI, and gene × BOLD-signal). Correcting for multiple comparisons, we considered a significance level of *p* = 0.017 (i.e., *p* = 0.05 divided by the number of interaction terms).

Finally, we formally described and compared different combinations of our predictors using model comparison. With an exhaustive search for all combinations between our predictors we wanted to find the best model explaining gIQ. We choose to compare a set of all combinations, resulting in 15 models. We performed model comparison based on difference in Bayesian information criterion (BIC), which is known to penalize for models with larger numbers of parameters more strongly, resulting in a parsimonious model.

All probability values for the abovementioned tests are reported non-directional (two-tailed).

If not stated differently statistical tests were performed using R version 3.2.4

## Results

### Association between IQ and polygenic scores

**Fig. 2:**
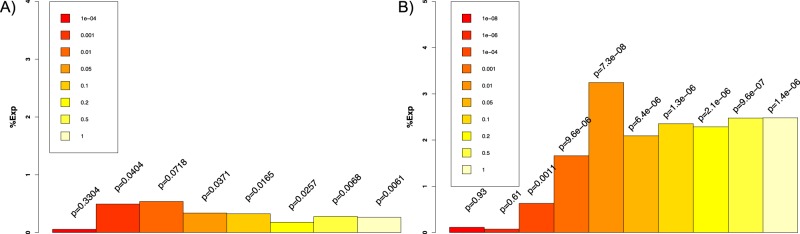
Variance explained (%Exp) of two different polygenic scores predicting general IQ. Each bar represents variance explained for a given set of multi-SNP predictors at a given *p*-value threshold that is color-coded. The color code is described in the legend within the plot and represents *p*-value thresholds for inclusion of SNPs. On top of the bars *p*-values for association with gIQ for each polygenic score are reported. **a** The left panel shows the polygenic score derived from Benyamin et al.^30^. **b** The right panel shows the polygenic score derived from Sniekers et al.^4^

With respect to our first research question regarding the influence of genetics on gIQ, we observed that the polygenic score by Benyamin et al.^30^ at a *p*-threshold of 0.1 comprising 16 972 SNPs was significantly associated with gIQ (0.33% variance explained, degrees of freedom (df) = 1376, *p* = 1.7 × 10^−2^; Fig. 2a, and Table 2 in SI).

**Fig. 3:**
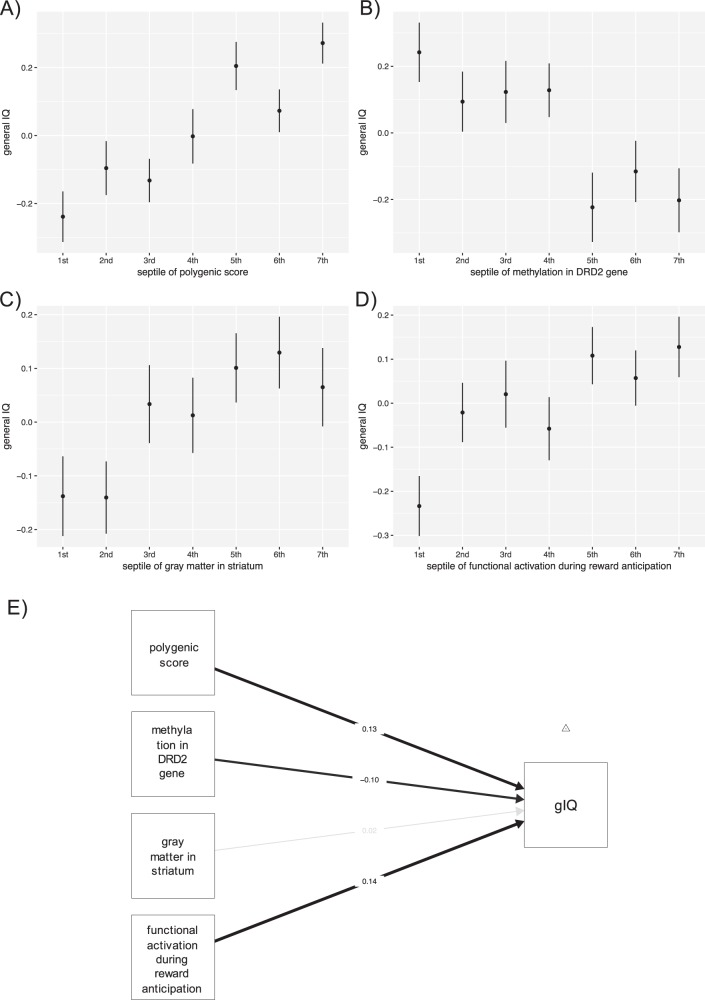
Candidate markers predicting general IQ. For display purpose, we grouped individuals into septiles of the candidate markers and plotted the mean phenotypic value (here general IQ) for each quantile on the *y*-axis^52^. Error bars indicate standard error of the mean. **a** General IQ can be predicted using polygenic score from Sniekers et al.^4^ at a *p*-threshold of 0.01 comprising 5636 SNPs explaining 3.2% of variance (df = 1376; *p* = 7.3 × 10^−8^; correcting for age, gender, study site, principal components from imputation, and genetic strata). **b** Here we display association with the marker with the lowest *p*-value (methylation count in dopamine D2-receptor gene, *DRD2* cg26132809) among our candidate markers. We grouped individuals into septiles of their methylation level (higher septile rank indicating higher probability of methylation) and plotted those septiles against mean general IQ score on the *y*-axis. General IQ is negatively correlated with candidate marker for dopamine neurotransmission in our regression model (2.7% of variance explained, df = 803, *p* = 3.18 × 10^−4^ correcting for age, gender, study site, wave information, and variability in cell type) indicating that higher methylation count, which is considered as downregulation of transcription of DRD2 receptor, is related to lower IQ scores. **c** Gray matter density in bilateral striatum was used to group individuals into septiles. We plotted gray matter density against general IQ and found 0.71% variance explained (df = 1399, *p* = 1.7 × 10^−3^), correcting for age, gender, site, and total brain volume. **d** Here we plot general IQ by reward anticipation signal (BOLD-signal) in region of interest (ROI). We grouped individuals into septiles of beta parameter estimates (BOLD-signal) and plotted mean general IQ for each quantile on the *y*-axis for display purposes. General IQ is positively correlated with functional activation of the ventral striatum (1.4% of variance explained, df = 1463, *p* = 4.11 × 10^−6^; correcting for gender, age, and study site). **e** Regression model illustrating neurobiological correlates of general IQ in an overlapping sample of *n* = 755. A multiple linear regression model with general IQ (gIQ) as outcome variable was estimated with the residuals of the following predictors: polygenic score (from Sniekers et al.), methylation in *DRD2* gene, gray matter in striatum, and functional activation during reward anticipation. The whole model was significant with an adjusted *R*^2^ = 0.04 (df = 750, *p* = 3.3 × 10^−7^). On the edges, we display the standardized parameter estimates for each predictor (beta) describing how many standard deviations the dependent variable (gIQ) will change, per standard deviation increase in the predictor variable. With respect to the different predictors, we could replicate previous findings that the established polygenic score (including 5636 SNPs significant at a *p*-threshold of 0.01) shows an association with general IQ (beta = 0.13, *p* = 2.8 × 10^−4^). We find variance in methylation count in our candidate CG site (*DRD2* cg26132809) that is negatively associated with general IQ (beta = −0.10, *p* = 6.2 × 10^−3^), indicating that higher methylation (lower gene activity) being associated with lower gIQ. In this subsample gray matter density in striatum was not associated with gIQ (beta = 0.02, *p* = 0.5). BOLD-signal change during reward anticipation significantly predicts cognitive capacity (beta = 0.14, *p* = 9.4 × 10^−05^)

With respect to the score provided by Sniekers et al., we found the maximal proportion of variance explained with 3.2% (*p* = 7.3 × 10^−8^, df = 1376, bootstrapped confidence interval (CI): 1.64–5.43%) using a score comprising 5636 SNPs significant at a *p*-threshold of 0.01 (Figs. 2b and 3a, and Table 2 in SI).

### Association between IQ and epigenetic markers of dopaminergic neurotransmission

Regarding our second research question, we found significant effects of methylation on gIQ in a CG site in the *DRD2* gene (cg26132809), which survived Bonferroni correction for multiple testing (2.7% variance explained, df = 803, *p* = 3.18 × 10^−4^, bootstrapped CI: 0.01–2.94%; Figs. 1a and 3b, and Table 3 in SI).

### Association between IQ and gray matter density in striatum (MRI)

We found a positive correlation between gray matter density in bilateral striatum with gIQ (0.71% variance explained, df = 1399, *p* = 1.7 × 10^−3^, bootstrapped CI: 0.09–1.87%; Fig. 3c). Using split-half cross-validation, we could confirm this finding (*d*_0_: 0.81%, df = 698, *p* = 2.2 × 10^−2^; *d*_1_: 0.64%, df = 699, *p* = 3.2 × 10^−2^).

### Association between IQ and ventral striatal BOLD-signal (fMRI)

In accordance with our third research question, we found that in our ROI, the ventral striatum, beta parameter estimates of reward anticipation (BOLD-signal) showed a significant positive association with gIQ (1.4% variance explained, df = 1463, *p* = 4.1 × 10^−6^, bootstrapped CI: 0.49–3.24%; Fig. 3d). This finding was confirmed using split-half cross-validation (*d*_0_: 1.4%, df = 726, *p* = 1.1 × 10^−3^; *d*_1_: 1.7%, df = 725, *p* = 4.4 × 10^−4^).

In an exploratory analysis we also observed that gIQ was positively correlated with functional activation during reward anticipation (BOLD-signal) in a large network outside of the ventral striatum as well in frontal and temporal regions (see Fig. 2 and Table 6 both in SI).

### Influence of genetics, epigenetics, gray matter, and striatal activation on IQ

Calculating one regression model with residuals of our candidate markers, including the polygenic score (by Sniekers et al.), epigenetic finding (*DRD2* cg26132809), gray matter in striatum, and striatal activation, we observed that an increase in the polygenic score of one SD leads to a beta = 0.13 change in gIQ (*p* = 2.8 × 10^−4^; Fig. 3b). Increase in methylation count in candidate CG site (*DRD2* cg26132809) was associated with a decrease in gIQ (beta = −0.1, *p* = 6.2 × 10^−3^). There is a positive effect of BOLD-signal change during reward anticipation predicting gIQ (beta = 0.14, *p* = 9.4 × 10^−5^). In this additional analysis gray matter density in striatum showed no significant association with gIQ (beta = 0.02, *p* = 0.5; Fig. 3b). Calculating a correlation matrix, we found no significant association between the neurobiological predictors (Table 1). Exploring possible non-additive effects, we found no significant interaction between the polygenic scores (Snieker et al. and Benyamin et al.) and our candidate markers (epigenetics, gray matter, and striatal activation). We conducted an exhaustive search for possible combinations of predictors and found the lowest BIC for a model comprising polygenic score by Sniekers et al., methylation count in the *DRD2* gene (cg26132809) and BOLD-signal during reward anticipation in the ventral striatum (Table 2). This model explained 4.81% of variance (*p* = 1.05 × 10^−7^, df = 751, bootstrapped CI: 2.22–9.04%). We calculated an additional correlation matrix in order to explore our candidate markers association with WISC IV subscales (see supplementary Table 7).

**Table 2:** Top six models of model comparison, among all possible combinations of 15 models

Model	∆BIC	df
gIQ ~ polygenic score + epigenetic + BOLD	0	751
gIQ ~ polygenic score + BOLD	1.02	752
gIQ ~ polygenic score + epigenetic + BOLD + gray matter	6.16	750
gIQ ~ epigenetic + BOLD	6.73	752
gIQ ~ polygenic score + BOLD + gray matter	7.08	751
gIQ ~ BOLD	8.24	753

From top to bottom we display the models starting with the lowest Bayesian information criterion (BIC). We used the overlapping sample of *n* = 755 and residuals of our predictors (partialling out variance from variables of no interest)

*df* degrees of freedom, *gIQ* general IQ

∆BIC, difference in Bayesian Information Criterion compared to the best model: ∆BIC = 0; BOLD, functional activation during reward anticipation in striatum; epigenetic, methylation in CG site of DRD2 gene cg26132809; polygenic score, polygenic score including 5636 SNPs significant at a *p*-threshold of 0.01 from Sniekers et al.; gray matter, gray matter density in striatum

## Discussion

Individual differences in intelligence have a substantial heritable background, while strong increases in test performance across the world in the last decades also point to strong environmental effects^7,46^. Using a polygenic score previously associated with cognitive capacity in children^30^ and a novel score^4^ tested in children and adults, we were able to replicate significant associations with gIQ, with the score based on 78 308 adults^4^ performing better than the one based on a sample of 17 989 children^30^. The striking difference between heritability estimates derived from twin and adoption studies (around 50–70%^2,3^) and variance explained by common genetic polymorphisms (around 5%^5^) for many traits has been labeled “the case of the missing heritability”. For example, regarding the partially heritable and polygenic human trait “body height”, polygenic scores account for 10% of the variance^47^, much below the high heritability estimates derived from monozygotic twin studies (heritability estimates around 85%^3^). So the discrepancy between variance explained by polygenic risk scores and twin studies may simply be due to the fact that polygenic risk scores only include common genetic polymorphisms and do not assess effects of rare gene variants. On the other hand, if epigenetic variation is transmitted to the offspring, as has been shown for some stress-related epigenetic effects^28^, a more or less substantial part of the presumably genetic background regarding IQ test results may indeed be due to epigenetic factors (and hence environmental effects including social exclusion or discrimination stress).

Searching for neurobiological markers associated with dopaminergic neurotransmission in light of studies linking this system to cognitive capacity^11,17,18^ we found significant associations between methylation of *DRD2* gene (cg26132809) and gIQ. Epigenetic control of gene expression is modulated by environmental factors such as stress exposure to the individual or in some cases parental generation^48^. Stress exposure and further environmental factors also strongly modulate dopaminergic neurotransmission, with relations to epigenetic modification unexplored. In line with previous findings^11^ we found gIQ to be related to gray matter density in striatum. These observations suggest a striatal contribution to the malleability of cognitive capacity^49^.

The association between ventral striatal activation and gIQ was found to be robust using split-half cross-validation as well as estimation of bootstrapped confidence intervals. In the MID task, temporarily unpredicted presentation of reward-associated stimuli elicit functional activation of the ventral striatum (BOLD-signal), which was previously associated with dopamine release measured indirectly by displacement of radio ligands of dopamine D2 receptors in this brain area^21^. Unlike in studies directly quantifying the size of the reward prediction error using computational modeling^31,50^, in the MID task, the size of the temporal error in the prediction of reward-anticipatory cues cannot be individually computed. Although not limited to the ventral striatum, finding the strongest effect in this region suggests that dopamine-dependent encoding of reward-anticipatory cues and prediction errors contribute to cognitive flexibility and rapid decision-making.

Calculating a regression model in a subsample of 755 subjects with fully available data for all predictors we found polygenic score of Sniekers et al.^4^, epigenetic markers of the *DRD2* gene and the ventral striatal BOLD-signal were significantly associated with gIQ with a similar effect size. In combined assessment in this subsample, gray matter density in striatum did not show a significant effect. Exploring the interrelatedness of our candidate markers, we found no significant association, thus pointing to rather independent sources of variance for gIQ. The abovementioned polygenic scores (Sniekers et al. and Benyamin et al.) did not show a significant interaction effect with our candidate markers (epigenetics, gray matter and striatal activation) in our sample. In order to formally describe and compare possible models that predict gIQ, comparison of all possible combinations of our predictors resulted in a model comprising BOLD-signal during reward anticipation in the ventral striatum, methylation count in the *DRD2* gene and the polygenic score. Altogether, the winning model points to a rather independent contribution of variance of dopaminergic neurotransmission to variance in gIQ on the one hand and genetic differences on the other hand.

Limitations of our study include the sample size, which is rather large for neuroimaging studies but exceedingly small for explorative genetic and epigenetic studies. This is reflected in rather large CIs when applying bootstrap procedures in epigenetic markers. Furthermore, our sample size is too small for the detection of epigenome-wide markers. Therefore, the DNA methylation score was limited to CG sites in selected dopaminergic genes, with only a single CG site emerging as significant. It is highly likely that a more comprehensive DNA methylation analysis would have identified more epigenetic loci, which are associated with IQ score. Despite the relatively small sample size, we were able to replicate effects of polygenic score on gIQ derived from large samples^4,30^. Further limitations include that our epigenetic markers are assessed in peripheral blood. They may not reflect variance in brain tissue and have to be validated in studies with methods directly accessing tissue in the central nervous system^51^. The cross-sectional design of the study does not allow any statement concerning causality. Further studies with a longitudinal design in possible quasi experimental settings are warranted.

Taken together, our findings suggest that both functional activation of the reward system, epigenetic control of dopaminergic neurotransmission, and genetic markers contribute to gIQ. Of note, the effect sizes studied are small but in the same range as previous studies (2.4%^10^ in previous structural imaging studies and up to 4.8%^4^ for previous polygenic scores). Eventually, it is fundamental for the understanding of cognitive capacity that we find variable neurobiological correlates of gIQ. Variance of methylation count in our epigenetic candidate marker and individual differences in ventral striatal activation during reward anticipation seem to be independent predictors and do not show a relation with genetic correlates. Observing an association between epigenetic markers and neural signatures of gIQ should encourage further studies exploring mechanisms that mediate genetic and environmental effects on the neurobiological correlates of cognitive functions.

## Electronic supplementary material

Supplemental material

Sup fig 5

sup fig 4

sup fig 3

sup fig 2

sup fig 1
