# Supplementary material for: Epigenetic variance in dopamine D2 receptor: a marker of IQ malleability?
Source: Transl Psychiatry. 2018 Aug 30;8:169. doi: 10.1038/s41398-018-0222-7 (PMC6117339; doi:10.1038/s41398-018-0222-7)
Supplement: Supplementary file 1 — Supplemental material [file 41398_2018_222_MOESM1_ESM.docx]

Supplemental Information for

**Epigenetic variance in dopamine D2-receptor: a marker of IQ malleability?**

**Intelligence measures**

A neuropsychological test battery was assessed^1^. According to the Technical Report for the WISC-IV^2^ the General Ability Index (GAI) provides a composite score that is based on three Verbal Comprehension and three Perceptual Reasoning subtests and does not include the Working Memory or Processing Speed subtests. The latter tests are included in the Full Scale IQ (FSIQ). According to the above mentioned manual it has been discussed whether or not to include working memory components to general IQ scores. More recent findings support the notion that working memory and processing speed do contribute substantially to general cognitive ability^3,4^. We assessed a general cognitive ability score based on the following available WISC-IV scores: matrix reasoning, block design, digitspan backward and forward, similarities and vocabulary. Principal component analysis was performed using each of the six cognitive measures and the first principal component was used for further analyses as marker of general cognitive capacity (see Table 1).

**Genetics**

Data was collected from eight different study sites and genotyped in three batches which constitute subsamples that were quality checked, imputed and analyzed separately according to standards from the Psychiatric Genomics Consortium (PGC). The quality control parameters for retaining SNPs and subjects were: SNP missingness <0.05 (before sample removal); subject missingness <0.02; autosomal heterozygosity deviation (| F_het_ | <0.2); SNP missingness <0.02 (after sample removal); difference in SNP missingness between cases and controls <0.02; and SNP Hardy-Weinberg equilibrium (*P*>10^−6^ in controls or *P*>10^−10^ in cases).

Genotype imputation was performed using the pre-phasing/imputation stepwise approach implemented in IMPUTE2 / SHAPEIT (chunk size of 3 Mb and default parameters). The imputation reference set consisted of 2,186 phased haplotypes from the full 1000 Genomes Project dataset (August 2012, 30,069,288 variants, release “v3.macGT1”). After imputation, we identified SNPs with very high imputation quality (INFO >0.8) and low missingness (<1%) for further quality control. After linkage disequilibrium pruning (r^2^>0.02) and frequency filtering (MAF >0.05), there were 77,385 autosomal SNPs across all three datasets of European ancestry. This SNP set was used for robust relatedness testing and population structure analysis. Relatedness testing was done with PLINK; pairs of subjects with $\hat{\pi}$ >0.2 were identified and one member of each pair removed at random after preferentially retaining cases over controls. Principal component estimation was performed with the same collection of autosomal SNPs. We tested the first 20 principal components for phenotype association (*using* logistic regression with study indicator variables included as covariates) and evaluated their impact on the genome-wide test statistics using λ. Six principal components namely 1,2,3,4,5,7 were included in all association analyses. The final analysis consisted of 9,175,717 SNPs for the score according to Benyamin et al.^5^ and 8,914,280 SNPs for the score established by Sniekers et al.^6^. We predicted gIQ using the obtained polygenic scores (see Table 2). For computation of scores according to Benyamin et al. there was no SNPs associated with gIQ at P-thresholds p=5x10^-8^ and P=1x10^-6^, therefore no polygenic score could be established at those P-vale thresholds.

**Epigenetics**

DNA was extracted (by the Centre National de Génotypage in Paris —Mark Lathrop’s lab) from whole blood samples (∼10mL) collected at the age of 14 and preserved in BD Vacutainer EDTA tubes (Becton, Dickinson and Company) using the Gentra Puregene Blood Kit (QIAGEN) according to the manufacturer’s instructions. DNA samples were processed and hybridized in two waves.

After removal of samples with insufficient DNA (N=14), duplicates (samples for which DNA was send both in the first and in the second wave, N=6) and mislabeled probes (during DNA extraction, N=1), quality control and normalization was performed on DNA methylation from N = 1287 individuals.

Preprocessing of raw methylation were performed in two waves. Raw DNA methylation signals were computed into β values [β = (methylated signal)/(methylated +unmethylated signals) at each locus]. These methylation ratios were transformed by logit transformation, as such transformation has been found to show heteroscedasticity^7^. Within- and between- sample normalization was performed using the stratified quantile normalization implemented in the Bioconductor minfi package^8^.

Preprocessing of raw microarray intensities into usable methylation measurements was performed on the combined sample. Detecting and removing low-quality samples has been shown to improve downstream results.

Before proceeding to data analysis, quality control was performed using the minfi package^8^. Bad samples were removed by using the removeBadSamples argument in the preprocessQuantile function of the minfi package. Briefly, the median methylated and median unmethylated signal for each sample were calculated and the samples where their average falls below the default badSampleCutoff were removed (all samples from wave 1 and wave 2 passed this QC step). All probes, including those containing SNPs that might affect the measurement of DNA, were kept.

Additionally, information on sex was estimated by using getSex function in the minfi package, which estimates samples sex based on the methylation values on the sex chromosomes. This estimated sample sex was verified with reported gender information. One subject showed gender data that did not match the reported gender information. This individual was removed from downstream analyses.

The samples were hybridized and processed in two different waves. Global methylation measurements were found to be different between those two batches. Thus, we had to include the experimental wave information as covariate in further analyses.

Much variability in methylation level can be accounted for by cell heterogeneity^9^. For each individual, cell counts for the 6 major cell types present in blood (i.e., CD8^+^ cytotoxic T-cells, CD4^+^ helper T-cells, natural killer cells, B-cells, monocytes and granulocytes) were estimated using the estimateCellCounts function in the minfi package. This function obtains sample-specific estimates of cell proportions based on reference information on cell-specific methylation^10^ and has been recently shown to be robust and computationally effective in reducing false positives^11^. The first two principle components of estimated differential cell counts were utilized in downstream analysis to correct for variance from cell type.

**Task details**

The subject underwent a modified version of the MID task. The subject could tell where the target will appear and how many points there were to be won by means of the (reward indicating) cue symbol that appeared on screen before each trial (see Figure 1). Task cues had different shapes: a triangle meant no points, a circle with a line meant two points and a circle with three lines meant ten points. The cue was followed by a variable anticipation interval (4000-4500 milliseconds). Responding too early or too late would result in a loss. The task lasts 11 minutes and is adaptive. The maximum that can be won is 200 points. Subjects received one M&M (or similar chocolates/sweets) for every five points won to enhance motivation during the task.

To achieve a correct response rate of 66% for all participants, the duration of target presentation was adapted to individual performance. The target was presented between 250-400 millisecond; the adjustment was effectuated in 10 millisecond steps if the participants performed better or worse than a 66% hit rate. The target time remained the same if the success rate was exactly 66%^12^. During the feedback phase, the amount of points won by participants in the respective trial and the total amount won so far was presented (1450 milliseconds). The intertrial interval was jittered (3500-4150 milliseconds). Before performing the task in the scanner, participants trained the task to ensure that the association of cues and feedback was learned. In order to keep the task short and simple no loss trials were included. Reaction times were measured as the time between cue presentation and button hit. Subjects with a reaction time deviating more than 3.5 SD were excluded from further analysis because they were considered to engage insufficiently in the task, resulting in a sample size of 1475 subjects.

**Structural MRI**

Automated structural MRI data processing was performed using voxel based morphometry as implemented in the VBM8 toolbox (Structural Brain Mapping Group, University of Jena, Germany; http://dbm.neuro.uni-jena.de/vbm8) for SPM8. The origin of the images was manually set to the anterior commissure to facilitate the subsequent normalization on the Montreal Neurological Institute (MNI) template. Subjects’ T1 images were manually inspected and 1401 showed sufficient quality criteria and entered further analysis. Individual tissue probability maps of gray matter, white matter and cerebrospinal fluid were automatically identified and registered into stereotactical standard MNI space. To increase the accuracy of inter-subject alignment, this was based on a sample customized template computed by a diffeomorphic image registration algorithm (DARTEL) developed by Ashburner^13^. We obtained relative volumes that are the Jacobian determinants of the deformation field from warping the individual brain. To preserve the amount of gray matter density, we multiplied the partitioned images by the relative voxel volumes. The images were then spatially smoothed with an isotropic Gaussian kernel of 8mm.

For further analysis, total intracranial volume was taken into account. We calculated total brain volume by summing up the volumes of the segmented images of gray matter, white matter and cerebrospinal fluid.

**Functional MRI**

Functional data were acquired using a gradient-echo-planar-imaging sequence (echo time 30 milliseconds, repetition time 2.2 seconds, flip angle 75°). 300 volumes were obtained, each consisting of 40 slices (2.4 mm thickness. 1 mm gap, voxel size: 3.4 mm× 3.4 mm × 3.4 mm). Slices were aligned to the AC-PC-Plane.

Pre-processing and statistical analysis for blood-oxygen-level-dependent (BOLD) responses were performed using statistical parametric mapping software (SPM8; Wellcome Trust Centre for Neuroimaging, UCL, London, UKM <http://www.fil.ion.ucl.ac.uk/spm)>. Subjects’ individual fMRI-images were slice-time corrected using the middle slice as the reference. All slices were spatially realigned, resliced, and nonlinearly warped on Montreal Neurological Institute space using a custom echo-planar imaging (epi) template. This custom-made template was created on the mean of a set of echo planar images of 240 randomly selected subjects (30 for each imaging site). Data were smoothed with a 5-mm Gaussian filter.

The following regressors were used for constructing a first-level model for each individual subject: anticipation of big, small and no rewards and feedback for big, small and no rewards. An additional regressor for all targets was added. All modelled events were convolved with SPM’s canonical hemodynamic response function. Movement parameters were included as covariates for each subject (three translational and three rotation parameters). Contrast images were created for each subject. We found a robust main effects of anticipation of *big and small win vs. no win* in the ventral striatum^14^ with a maximum in the ventral striatum (MNI coordinates 9 8 1 p=FWE-corr.<0.05; Table 3). The above mentioned contrast activated a large cluster extending from its maximum in the ventral striatum to frontal, parietal, visual cortex as well as cingulate cortex. The association of the BOLD-signal during reward anticipation and general IQ had its maximum in striatum (MNI coordinates -9 8 1; pFWE-corr=1.62x10^-4^) and was found to be significant in a variety of regions (Figure 2 and Table 4)

**Model comparison**

Given different models, we wanted to know which of the model is the best, and we wanted to compare the models accordingly. We choose to use Bayesian information criterion (BIC) for comparing models, because we wanted to compare models with different numbers of parameters. From a variety of indices for model comparison and in light of our rather large n and different numbers of parameters, we choose BIC, because it tends to penalize for more parameters more strongly.

**Association between SNPs at relevant CG-site *DRD2* cg26132809, methylation and gIQ**

According to a reviewer’s request, we performed an exploratory analysis for interrelatedness between methylation in our candidate site (*DRD2* cg26132809) and underlying SNPs in that area. We explored SNPs that are 50kbp up-and downstream from our gene of interest and calculated linear regression models predicting methylation at CG-site *DRD2* cg26132809. We included age, gender, principal components from imputation and genetic strata, as variables of no interest to correct for possible confounding effects.

The analysis revealed 392 SNPs that are highly correlated, therefore we choose a rather liberal p value threshold of p<0.001. We found that 11 SNPs 50kbp up- and downstream from DRD2 receptor CG-site cg26132809 are related to methylation at that CG-site (Supplementary Figure 3), additionally they are highly related with each other (Supplementary Figure 4). This is not surprising given the nature of direct dependence of methylation probability and underlying base pairs.

For testing the notion that SNPs in that regions directly show an association with gIQ we explored all 392 SNPs 50kbp up- and downstream from *DRD2* receptor CG-site cg26132809. Even at a very liberal p-value threshold of p<0.001 we found no relevant SNPs (Supplementary Figure 5).

**Gene features and association between methylation count in blood and brain tissue**

For a more grounded interpretation of blood-based methylation results, we checked our candidate markers in the BECon tool^15^. The tool provides information on CG-site features, variability and association of DNA methylation in blood cells and brain tissue. Notably, it provides information about the location of the CG-site (i.e. intergenic, intragenic, promotor region), the variability (if meaningful methylation should show variability), correlation coefficient with methylation in the three brain areas and variance depending on cell composition. Although this tool does not provide associations with our region of interest, the striatum, it provides a comprehensive approach to the association of markers identified in the peripheral blood for complex traits that probably manifest their impact in the brain. According to the BECon tool, our candidate methylation site that showed a significant association with gIQ (cg26132809) lies within the promoter of DRD2 and shows sufficient variability (>0.05 beta value) in BA10 (frontopolar prefrontal cortex), BA20 (temporal cortex) as well as in the blood (see electronic supplementary table 1). Considering variability, and looking at methylation of DNA in the blood and the brain at cg26132809, the sample shows a very weak negative association with BA10 (frontopolar prefrontal cortex) and a negative association rho=-0.28 in BA20 (temporal cortex). Of course, there is heterogeneity in cell type composition that accounts for variance in methylation, therefore we corrected for cell composition in the association analysis.

***DRD* *3,4* and *5* and their relation to gIQ**

Dopamine receptors are grouped in two families. D1-like (D1 and D2 receptors) and D2-like receptors (D2, 3 and 4). They are characterized by differential expression patterns throughout the brain. D1 and D2 receptors are the most abundant receptors with expression in regions relevant for motor, limbic and neuroendocrine functioning (for a review see Meador-Woodruff 2000^2^). D3, D4 and D5 mRNA are one to two orders of magnitude lower than that of the D1 or D2^3,4^. Therefore our a priori hypothesis did not include D3, 4 and 5 receptor subfamily CG-sites. According to a reviewer’s request, we performed exploratory tests for an association with gIQ for cg-sites near D3, D4 and D5 receptor gene. There was no CG-site within the search frame for DRD4 gene. Applying Bonferroni correction (p-value=0.05/6 CG-sites: p<8.3x10^-3^) for multiple testing, no significant CG-site could be determined (see supplementary Table 6).

**Association between candidate markers and WISC-IV subscales**

According to a reviewer’s request we explored possible differential associations between biological candidate markers and WISC IV subscales (see Table 7). Based on residuals of candidate markers (polygenic score (from Sniekers et al.), methylation in DRD2 gene, gray matter in striatum and functional activation during reward anticipation) we explored possible differential associations with WISC-IV subscales in an overlapping sample of n=755 (see Table 7). The effect sizes are very small. We feel we can only very carefully interpret the differential associations, as possible evidence for different sources of variance being captured by our biological candidate markers: The correlation matrix shows associations between polygenic score (from Sniekers et al.) and the following WISC IV subscales: vocabulary, similarities and block-design. Those subscales cover a variety of domains including crystalized intelligence as well as non-verbal reasoning. Concerning our candidate epigenetic marker (methylation in CG-site DRD2 cg26132809) we found associations with subscales similarities and digitspan forward. Those subscales capture crystalized intelligence, working memory and attention. The BOLD signal from reward anticipation shows significant associations with vocabulary, similarities, block-design and matrix-reasoning. This represents a wide variety of abilities from crystalized intelligence to non-verbal reasoning and fluid intelligence. Striatal gray matter volume did not show any significant associations with WISC IV subscales in this subsample.

Tables

**Table 1| Principal Component Analysis on WISC-IV subscales (n = 1475).**

|  | Comp.1 | Comp.2 | Comp.3 | Comp.4 | Comp.5 | Comp.6 |
| --- | --- | --- | --- | --- | --- | --- |
| vocabulary | -0.449 | 0.466 | -0.041 | 0.092 | 0.659 | -0.369 |
| similarities | -0.458 | 0.421 | -0.049 | 0.053 | -0.746 | -0.224 |
| blockdesign | -0.281 | -0.57 | 0.591 | 0.168 | -0.036 | -0.466 |
| matrixreasoning | -0.232 | -0.478 | -0.74 | -0.27 | -0.004 | -0.312 |
| digitspan forward | 0.493 | 0.224 | 0.158 | -0.586 | -0.055 | -0.578 |
| digitspan backward | 0.46 | 0.045 | -0.271 | 0.737 | -0.065 | -0.406 |
| cumulative variance explained | 0.49 | 0.714 | 0.864 | 0.958 | 1 | 1 |

**Table 2 Polygenic score according to Benyamin et al. 2014 and Sniekers et al. 2017 predicting general IQ in n=1388 subjects.** SNPs at different P-thresholds were included in order to build a polygenic score to predict the independent variable general IQ. Linear models included covariates for age, gender, principal components from imputation and genetic strata resulting in 1376 degrees of freedom. Significant p-value at p<0.05 are marked in bold, and correcting for the two different scores at p<0.025 are marked with an asterisk.

| Benyamin et al. | | | | | |  | Sniekers et al. | | | | | |
| --- | --- | --- | --- | --- | --- | --- | --- | --- | --- | --- | --- | --- |
| SNPs | *P*-threshold | coef | intercept | %Exp | p |  | SNPs | *P*-threshold | coef | intercept | %Exp | p |
| 0 | 0.00000005 |  |  |  |  |  | 12 | 0.00000005 | 0.064 | 0.124 | 0.113 | 0.93 |
| 0 | 0.000001 |  |  |  |  |  | 34 | 0.000001 | 0.221 | 0.093 | 0.075 | 0.61 |
| 54 | 0.0001 | 0.187 | 0.072 | 0.056 | 0.33 |  | 301 | 0.0001 | 0.591 | -0.099 | 0.631 | **1.1x10^-03^*** |
| 415 | 0.001 | 0.156 | 0.173 | 0.493 | **4.0x10^-02^** |  | 1149 | 0.001 | 0.461 | -0.119 | 1.661 | **9.6x10^-06^*** |
| 2765 | 0.01 | 0.065 | 0.075 | 0.538 | 7.2x10^-02^ |  | 5636 | 0.01 | 0.288 | -0.41 | 3.246 | **7.3x10^-08^*** |
| 10040 | 0.05 | 0.044 | 0.036 | 0.338 | **3.7x10^-02^** |  | 16609 | 0.05 | 0.16 | -0.136 | 2.093 | **6.4x10^-06^*** |
| 16972 | 0.1 | 0.041 | -0.023 | 0.325 | **1.7x10^-02^*** |  | 26104 | 0.1 | 0.147 | -0.242 | 2.355 | **1.3x10^-06^*** |
| 28100 | 0.2 | 0.032 | 0.021 | 0.177 | **2.6x10^-02^** |  | 40263 | 0.2 | 0.127 | -0.304 | 2.286 | **2.1x10^-06^*** |
| 51229 | 0.5 | 0.034 | -0.009 | 0.278 | **6.8x10^-03^*** |  | 66648 | 0.5 | 0.12 | -0.283 | 2.477 | **9.6x10^-07^*** |
| 70534 | 1 | 0.034 | -0.023 | 0.263 | **6.1x10^-03^*** |  | 86313 | 1 | 0.117 | -0.337 | 2.483 | **1.4x10^-06^*** |

**Table 3 Epigenetic effect on general IQ in n=817 subjects.** As covariates of no interest gender, age, site, first and second principal component of cell type as well as acquisition wave, were included in the model, resulting in 803 degrees of freedom. P-values that are significant correcting for multiple comparisons using Bonferroni correction p<2x10^-3^ are highlighted in bold and nominally significant effects p<0.05 are highlighted in italics.

|  | coef | Intercept | %Exp | p |
| --- | --- | --- | --- | --- |
| THcg05912121 | 2.095 | -1.73 | 0.006 | 2.15x10^-1^ |
| THcg08573687 | 3.41 | -3.112 | 0.153 | 1.88x10^-1^ |
| DDCcg01220564 | -0.431 | 0.338 | 0.616 | 7.12x10^-1^ |
| DDCcg13232821 | -0.771 | 0.668 | 0.047 | 4.08x10^-1^ |
| DDCcg04443360 | -0.418 | 0.409 | 0.351 | 7.04x10^-1^ |
| DDCcg20336341 | -0.751 | 0.584 | 0.004 | 5.26x10^-1^ |
| COMTcg11032634 | -0.587 | 0.037 | 0.007 | 3.82x10^-1^ |
| COMTcg03205258 | -0.333 | 0.034 | 0.028 | 5.90x10^-1^ |
| COMTcg03724721 | -0.176 | 0.223 | 0.525 | 9.40x10^-1^ |
| COMTcg25836061 | -1.125 | 1.039 | 1.245 | 5.68x10^-1^ |
| COMTcg12175949 | 0.346 | 0.049 | 0.005 | 5.35x10^-1^ |
| SLC6A3cg04210284 | 0.696 | 0.024 | 0.046 | 7.37x10^-1^ |
| SLC6A3cg04598517 | -0.163 | 0.082 | 0.009 | 9.36x10^-1^ |
| SLC6A3cg05030481 | -0.853 | 0.118 | 1.12 | 8.37x10^-1^ |
| SLC6A3cg12882697 | 0.562 | -0.038 | 0.431 | 6.86x10^-1^ |
| SLC6A3cg27037018 | 2.382 | -0.092 | 0.113 | 1.60x10^-1^ |
| DRD1cg18190187 | -3.732 | 0.249 | 1.254 | 5.18x10^-2^ |
| DRD1cg00698685 | -3.246 | 0.346 | 0.293 | 2.94x10^-1^ |
| DRD2cg26132809 | -3.972 | 1.004 | 2.703 | **3.18x10^-4^** |
| DRD2cg21330703 | -1.828 | 0.381 | 0.46 | 2.91x10^-1^ |
| DRD2cg16845257 | 0.121 | -0.04 | 0.017 | 9.35x10^-1^ |
| DRD2cg12176709 | -0.057 | 0.078 | 0.085 | 9.60x10^-1^ |
| DRD2cg20629239 | -3.581 | 0.598 | 1.378 | *1.24x10^-2^* |
| DRD2cg12758687 | -3.217 | 0.787 | 0.783 | 5.77x10^-2^ |

**Table 4 Main effect of anticipation signal.** Extent threshold: k = 30 voxels. We report p(FWE-corr.)<0.05, T-values (T), Z-score equivalent to T statistics (equiv Z), MNI coordinates as well as the region labelled according to individual Brain Atlases tool in SPM IBASPM 71 (region). Due to the strong anticipation effect and wide network that was found to be involved we report top 25 subpeaks in a vast cluster of k=40999 voxels. Gender, age and study site were included into the model as variables of no interest.

| peak |  |  | MNI coordinates | | |  |
| --- | --- | --- | --- | --- | --- | --- |
| p(FWE-cor) | T | equiv Z | x | y | z | region |
| <0.001 | 36.7531 | Inf | 9 | 8 | 1 | caudate nucleus |
| <0.001 | 36.5565 | Inf | -12 | 11 | -2 | caudate nucleus |
| <0.001 | 36.4611 | Inf | -9 | -19 | 7 | thalamus |
| <0.001 | 36.1154 | Inf | 15 | -91 | -5 | lingual gyrus |
| <0.001 | 35.8582 | Inf | 9 | -16 | 7 | thalamus |
| <0.001 | 35.1616 | Inf | -3 | 2 | 55 | med frontal gyrus |
| <0.001 | 35.0743 | Inf | -12 | -91 | -11 | lingual gyrus |
| <0.001 | 33.1176 | Inf | 9 | -22 | -5 | brain stem |
| <0.001 | 31.9067 | Inf | 42 | -19 | 61 | precentral gyrus |
| <0.001 | 31.7347 | Inf | -39 | -13 | 61 | precentral gyrus |
| <0.001 | 31.0693 | Inf | 39 | -10 | 61 | precentral gyrus |
| <0.001 | 29.6234 | Inf | -48 | -28 | 52 | postcentral gyrus |
| <0.001 | 26.9541 | Inf | -21 | -52 | -23 | cerebellum |
| <0.001 | 26.8962 | Inf | -30 | -7 | 52 | med frontal gyrus |
| <0.001 | 26.216 | Inf | 24 | -52 | -23 | cerebellum |
| <0.001 | 24.783 | Inf | -12 | -70 | 52 | superior parietal lobule |
| <0.001 | 24.5136 | Inf | 12 | -70 | 52 | superior parietal lobule |
| <0.001 | 24.1606 | Inf | -24 | -58 | 49 | superior parietal lobule |
| <0.001 | 23.5638 | Inf | 33 | 26 | 4 | inf frontal gyrus |
| <0.001 | 23.5238 | Inf | 0 | -52 | -20 | cerebellum |
| <0.001 | 22.9345 | Inf | 30 | -52 | 46 | superior parietal lobule |
| <0.001 | 22.8102 | Inf | 27 | -76 | -17 | lateral occipito temporal gyrus |
| <0.001 | 22.5969 | Inf | -3 | -31 | 25 | cingulate region |
| <0.001 | 22.0966 | Inf | -30 | 20 | 7 | insula |
| <0.001 | 21.9853 | Inf | -27 | -73 | 28 | superior parietal lobule |

**Table 5 Effect of general IQ on reward anticipation signal.** Extent threshold: k = 30 voxels. We report p(FWE-corr.)<0.05, T-values (T), Z-score equivalent to T statistics (equiv Z), MNI coordinates as well as the region labelled according to individual Brain Atlases tool in SPM IBASPM 71 (region). T-test for the effect for general IQ on the reward anticipation signal, controlling for gender, age and site.

| peak |  |  | MNI coordinates | |  |  |
| --- | --- | --- | --- | --- | --- | --- |
| p(FWE-corr.) | T | equiv Z | x | y | z | region |
| 1.24x10^-3^ | 5.286 | 5.260 | 27 | 50 | 25 | middle frontal gyrus |
| 5.56x10^-4^ | 5.450 | 5.422 | -33 | 47 | 25 | middle frontal gyrus |
| 2.83x10^-3^ | 5.111 | 5.088 | -33 | 44 | 37 | middle frontal gyrus |
| 4.39x10^-3^ | 5.016 | 4.994 | -36 | 35 | 46 | middle frontal gyrus |
| 3.74x10^-2^ | 4.512 | 4.496 | 18 | 26 | -8 | caudate nucleus |
| 1.85x10^-3^ | 5.203 | 5.178 | 18 | 17 | 1 | caudate nucleus |
| 7.78x10^-4^ | 5.382 | 5.355 | 12 | 11 | 4 | caudate nucleus |
| 1.62x10^-4^ | 5.691 | 5.659 | -9 | 8 | 1 | caudate nucleus |
| 4.52x10^-4^ | 5.491 | 5.462 | -15 | -1 | 13 | caudate nucleus |
| 1.26x10^-6^ | 6.551 | 6.502 | 39 | -7 | 49 | precentral gyrus |
| 3.77x10^-4^ | 5.527 | 5.498 | 0 | -16 | 13 | thalamus |
| 2.96x10^-3^ | 5.102 | 5.078 | 33 | -16 | -8 | putamen |
| 1.38x10^-3^ | 5.265 | 5.239 | 42 | -31 | 1 | temporal lobe |
| 8.27x10^-7^ | 6.620 | 6.570 | 15 | -55 | -8 | medial occipito temporal gyrus |
| 4.88x10^-6^ | 6.323 | 6.280 | -30 | -61 | -11 | medial occipito temp gyrus |

**Table 6 Epigenetic effect on general IQ in n=817 subjects for DRD3, DRD4 and DRD5.** As covariates of no interest gender, age, site, first and second principal component of cell type as well as acquisition wave, were included in the model, resulting in 803 degrees of freedom. There was no CG-site within the search frame for DRD4 gene.

|  | coef | Intercept | %Exp | p |
| --- | --- | --- | --- | --- |
| DRD3cg08479865 | 0.895 | -0.768 | 0.047 | 0.458 |
| DRD5cg26296488 | -0.301 | 0.076 | 0.056 | 0.5 |
| DRD5cg23847712 | -0.3 | 0.116 | 0.407 | 0.502 |
| DRD5cg03045635 | -0.894 | 0.205 | 0.093 | 0.166 |
| DRD5cg04597433 | -0.265 | 0.104 | 0.046 | 0.688 |
| DRD5cg06469345 | -0.522 | 0.083 | 0.249 | 0.511 |

**Table 7 Association between candidate markers and WISC IV subscales n=755.** We calculated pearsons correlation coefficients on residuals from our candidate markers with WISC IV subscales performance.

|  | vocabulary | similarities | block-design | matrix-reasoning | digitspan forward | digitspan backward |
| --- | --- | --- | --- | --- | --- | --- |
| polygenic score | 0.12** | 0.12** | 0.08* | 0.06 | 0.07 | 0.06 |
| epigenetic | -0.04 | -0.11** | -0.05 | -0.07 | -0.08* | -0.05 |
| BOLD | 0.08* | 0.12** | 0.11** | 0.13**** | 0.04 | 0.02 |
| gray matter | 0 | 0.05 | 0 | -0.03 | 0.05 | 0.05 |
| *Note: significant levels (2-tailed) p < .0001 ‘****’; p < .001 ‘***’, p < .01 ’**’, p < .05 ’*’*  *Abbreviations: BOLD (functional activation during reward anticipation), epigenetic (methylation in CG-site DRD2 cg26132809), polygenic score (polygenic score including 5636 SNPs significant at a P-threshold of 0.01 from Sniekers et al.), gray matter (gray matter density in striatum).* | | | | | | |

Figure legends

**Figure 1 Modified monetary incentive delay task and hypothesized phasic dopaminergic signal.** A) Cues indicating reward were presented. After the cue and a delay (4000-4500ms) subjects had to respond to a target. The delay between cue and target was adjusted to subject’s performance so that the percentage correct was held constant at 66%. The intertrial interval was jittered between 3500 and 4150ms. B) The hypothesized phasic dopaminergic signal that is related to the temporarily surprising occurrence of a reward predicting cue. This dopamine activation hypothetically corresponds to functional activation in the ventral striatum elicited by conditioned reward-indicating cues in the MID task. Figure modified and merged according to Schultz et al.^12^.

**Figure 2 Voxelwise test for associations of general IQ on effect of anticipation for the contrast big and small vs. no win in n=1475 subjects**. We observed significant associations comprising occipital, cerebellar, parietal, striatal, thalamic and frontal regions (p=FWE-corr <0.05 see Table 5). For display purposes the statistical map is thresholded at a minimum T-value of >2.5.

**Figure 3 Association between SNPs and methylation count in *DRD2* CG-site cg26132809 in n=817 subjects.** Plot of negative decadic logarithm of p-values for association of methylation count SNPs and 50kb pairs up- and downstream from *DRD2* CG-site cg26132809. We corrected for age, gender, principal components from imputation and genetic strata. The blue line marks significance threshold for p<0.001 and the red line p<0.05. Among 392 SNPs from 113359595 to 113525279 genomic coordinate (hg19), we identified 11 SNPs 50kbp up- and downstream from *DRD2* CG-site cg26132809 to be associated with methylation counts at *DRD2* CG-site cg26132809 p<0.001. We labeled top three SNPs within the plot.

**Figure 4 Association between top 11 SNPs SNPs associated with methylation at *DRD2* CG-site cg26132809.** Correlation matrix of top 11 SNPs identified from analysis of association of SNPs that reside within 50kb pairs up- and downstream from *DRD2* CG-site cg26132809 and are associated with methylation in *DRD2* CG-site cg26132809. Correlation coefficients are color coded with positive associations coded as dark blue.

**Figure 5 Association between SNPs 50kb pairs up- and downstream from *DRD2* CG-site cg26132809 and gIQ in n=817 subjects.** Plot of negative decadic logarithm of p-values for association of gIQ and SNPs 50kb pairs up- and downstream from *DRD2* CG-site cg26132809. We corrected for age, gender, principal components from imputation and genetic strata. The blue line marks significance threshold for p<0.001 and the red line p<0.05. Among 392 SNPs from 113359595 to 113525279 genomic coordinate (hg19), we identified no SNPs 50kbp up- and downstream from *DRD2* CG-site cg26132809 to be associated with gIQ at p<0.001.

Electronic supplementary tables

**Electronic supplementary table 1 Results from BECon tool for gene features and association between methylation count in blood and brain tissue for available CG-sites from candidate CG-sites for *TH*, *DDC*, *COMT*, *SLC6A3*, *DRD1* and *DRD2*.** CG-site codes (llumina identifier) are displayed, followed by chromosome number and genomic coordinate (hg19). Additionally, BECon provides information on genes associated with the CG-site (associated genes). The fifth column represents gene features that are associated to the CG-site. The following columns show correlation coefficients between methylation of peripheral blood and brain tissue (Cor Blood-…) followed by percentile ranks of correlations. The fields with correlation coefficients are marked in either light grey for coefficients >75% percentile ranks and in dark grey for >90% percentile ranks. The next two columns show variability that is to be accounted for by tissue type (delta betas between data unadjusted for cell composition and data adjusted for cell composition) and the according percentile ranks. The last five columns represent variability in methylation (light green sufficient variability beta >0.05).

**Electronic supplementary table 2 Results from BECon tool for gene features and association between methylation count in blood and brain tissue for available CG-sites from candidate CG-sites for *DRD3, DRD4* and *DRD5.*** CG-site codes (llumina identifier) are displayed, followed by chromosome number and genomic coordinate (hg19). Additionally, BECon provides information on genes associated with the CG-site (associated genes). The fifth column represents gene features that are associated to the CG-site. The following columns show correlation coefficients between methylation of peripheral blood and brain tissue (Cor Blood-…) followed by percentile ranks of correlations. The fields with correlation coefficients are marked in either light grey for coefficients >75% percentile ranks and in dark grey for >90% percentile ranks. The next two columns show variability that is to be accounted for by tissue type (delta betas between data unadjusted for cell composition and data adjusted for cell composition) and the according percentile ranks. The last five columns represent variability in methylation (light green sufficient variability beta >0.05).

References

1 Wechsler, D., Kaplan, E., Fein, D., Kramer, J., Morris, R., Delis, D., & Maelender A. *Wechsler intelligence scale for children: Fourth edition (WISC-IV)*. Pearson: San Antonio, TX, 2003.

2 Raiford SE, Weiss LG, Rolfhus E, Coalson D. General Ability Index WISC-IV Technical Report #4. San Antonio, 2008http://images.pearsonclinical.com/images/assets/WISC-IV/80720_WISCIV_Hr_r4.pdf.

3 Colom R, Rebollo I, Palacios A, Juan-Espinosa M, Kyllonen PC. Working memory is (almost) perfectly predicted by g. *Intelligence* 2004; **32**: 277–296.

4 Schweizer K, Moosbrugger H. Attention and working memory as predictors of intelligence. *Intelligence* 2004; **32**: 329–347.

5 Benyamin B, Pourcain Bs, Davis OS, Davies G, Hansell NK, Brion M-J *et al.* Childhood intelligence is heritable, highly polygenic and associated with FNBP1L. *Mol Psychiatry* 2014; **19**: 253–258.

6 Sniekers S, Stringer S, Watanabe K, Jansen PR, Coleman JRI, Krapohl E *et al.* Genome-wide association meta-analysis of 78,308 individuals identifies new loci and genes influencing human intelligence. *Nat Genet* 2017; **49**: 1107–1112.

7 Du P, Zhang X, Huang C, Jafari N, Kibbe WA, Hou L *et al.* Comparison of Beta-value and M-value methods for quantifying methylation levels by microarray analysis. *BMC Bioinformatics* 2010; **11**: 587.

8 Aryee MJ, Jaffe AE, Corrada-Bravo H, Ladd-Acosta C, Feinberg AP, Hansen KD *et al.* Minfi: A flexible and comprehensive Bioconductor package for the analysis of Infinium DNA methylation microarrays. *Bioinformatics* 2014; **30**: 1363–1369.

9 Jaffe AE, Irizarry RA. Accounting for cellular heterogeneity is critical in epigenome-wide association studies. *Genome Biol* 2014; **15**: 1–9.

10 Houseman EA, Accomando WP, Koestler DC, Christensen BC, Marsit CJ, Nelson HH *et al.* DNA methylation arrays as surrogate measures of cell mixture distribution. *BMC Bioinformatics* 2012; **13**: 86.

11 Mcgregor K, Bernatsky S, Colmegna I, Hudson M, Pastinen T, Labbe A *et al.* An evaluation of methods correcting for cell-type heterogeneity in DNA methylation studies. *Genome Biol* 2016; **17**. doi:10.1186/s13059-016-0935-y.

12 Whelan R, Watts R, Orr C a, Althoff RR, Artiges E, Banaschewski T *et al.* Neuropsychosocial profiles of current and future adolescent alcohol misusers. *Nature* 2014; **512**: 185–189.

13 Ashburner J. A fast diffeomorphic image registration algorithm. 2007; **38**: 95–113.

14 Büchel C, Peters J, Banaschewski T, Bokde ALW, Bromberg U, Conrod PJ *et al.* Blunted ventral striatal responses to anticipated rewards foreshadow problematic drug use in novelty-seeking adolescents. *Nat Commun* 2017; **8**: 14140.

15 Edgar RD, Jones MJ, Meaney MJ, Turecki G, Kobor MS. BECon: A tool for interpreting DNA methylation findings from blood in the context of brain. *Transl Psychiatry* 2017; **7**: 1–18.

16 Schultz W, Dayan P, Montague PR. A neural substrate of prediction and reward. *Science* 1997; **275**: 1593–1599.
