# Supplementary figures and images for: Epigenetic variance in dopamine D2 receptor: a marker of IQ malleability?
Source: Transl Psychiatry. 2018 Aug 30;8:169. doi: 10.1038/s41398-018-0222-7 (PMC6117339; doi:10.1038/s41398-018-0222-7)

# influence on general IQ

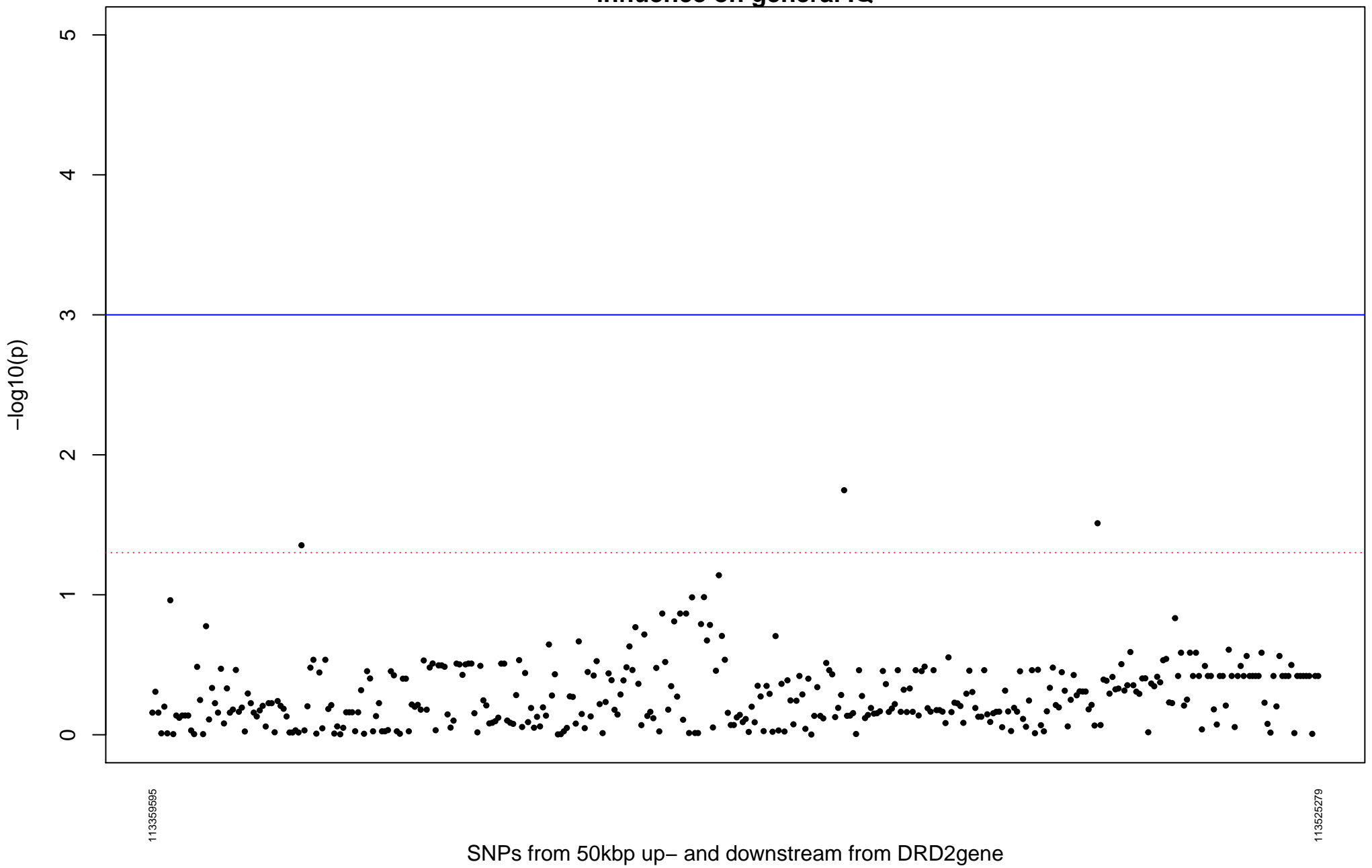

Supplement: Supplementary file 2 — Sup fig 5 [file 41398_2018_222_MOESM2_ESM.pdf]

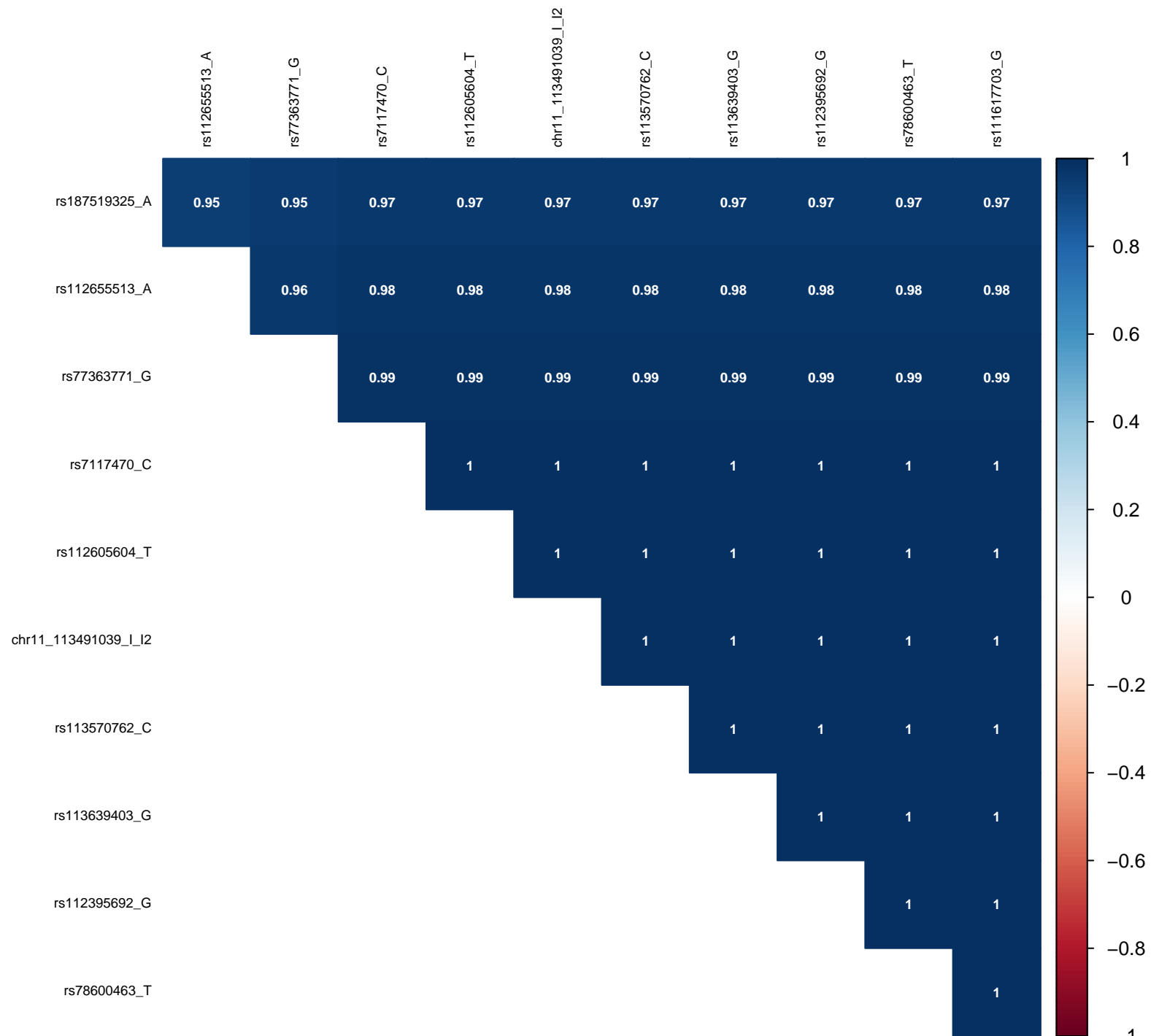

Supplement: Supplementary file 3 — sup fig 4 [file 41398_2018_222_MOESM3_ESM.pdf]

# influence on methylation DRD2\_cg26132809

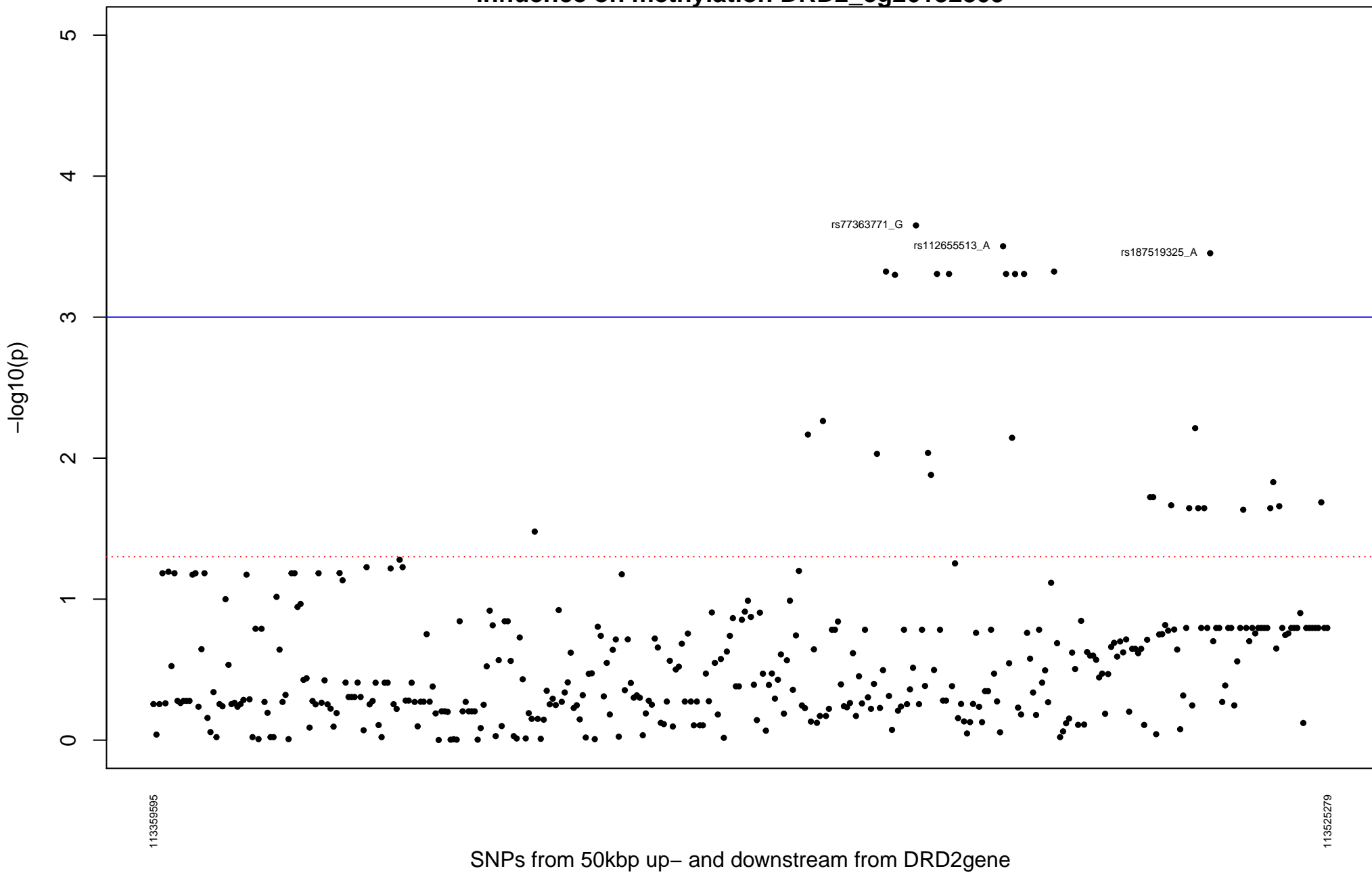

Supplement: Supplementary file 4 — sup fig 3 [file 41398_2018_222_MOESM4_ESM.pdf]

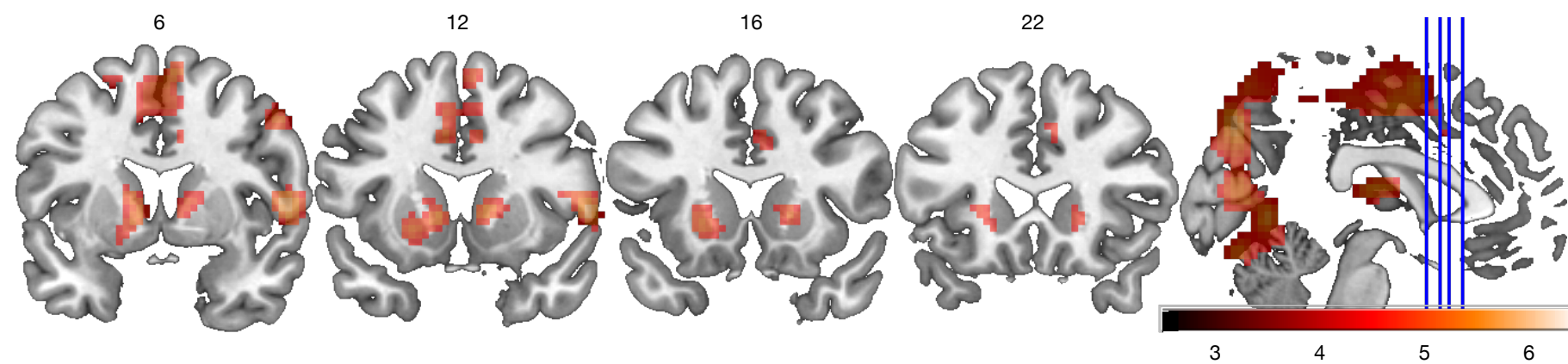

Supplement: Supplementary file 5 — sup fig 2 [file 41398_2018_222_MOESM5_ESM.pdf]

A)

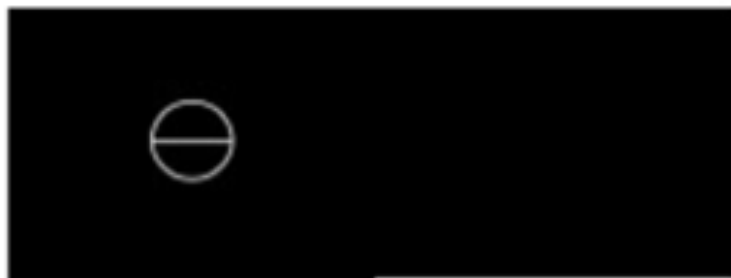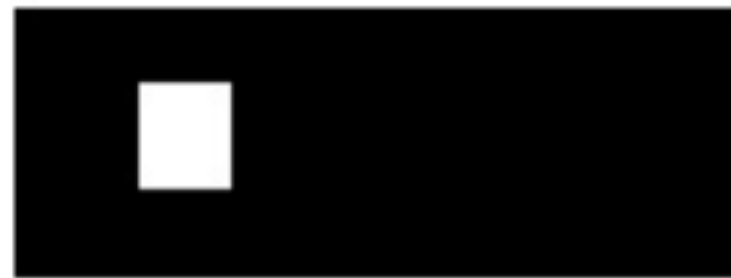

B)

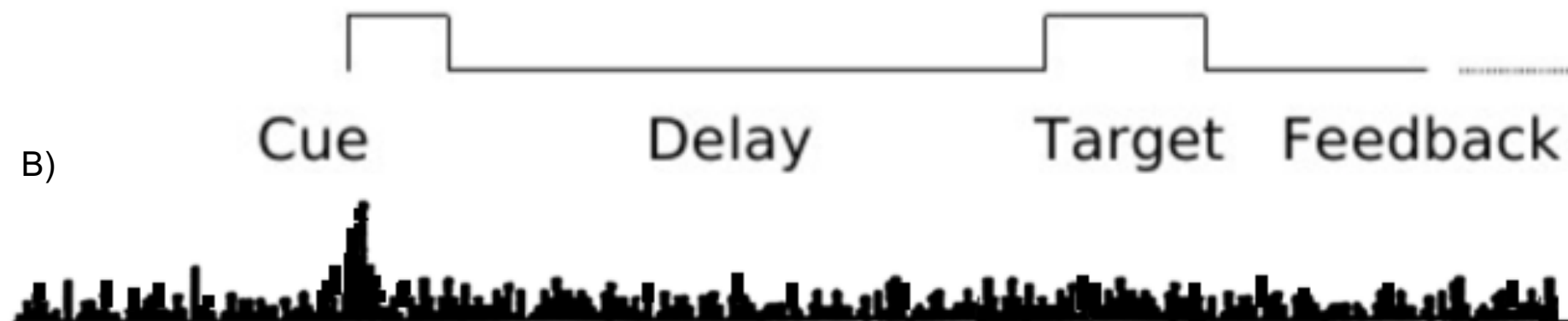

Supplement: Supplementary file 6 — sup fig 1 [file 41398_2018_222_MOESM6_ESM.pdf]
